# Human Papillomavirus and Epstein-Barr virus co-infection in Cervical Carcinoma in Algerian women

**DOI:** 10.1186/1743-422X-10-340

**Published:** 2013-11-19

**Authors:** Abdelhalim Khenchouche, Nabila Sadouki, Arab Boudriche, Karim Houali, Abdelaziz Graba, Tadamasa Ooka, Abdelmadjid Bouguermouh

**Affiliations:** 1 Departement de Microbiologie, Faculté des Sciences de la Nature et de la Vie, Université Sétif-1, Sétif, Algeria; 2 Institut Pasteur d’Algérie, Laboratoire Virus-Cancer, Sidi Fredj, Algeria; 3 Service de gynécologie, Hôpital de Zeralda, Algiers, Algeria; 4 Laboratoire LABAB, Faculté des Sciences Biologiques et Agronomiques, Université Mouloud Mammeri de Tizi-Ouzou, Tizi-Ouzou, Algeria; 5 Service de chirurgie-oncologie, Centre de Pierre et Marie Curie, CHU Mustapaha Bacha, Algiers, Algeria; 6 Laboratoire de Virologie Moléculaire, UMR5537, C.N.R.S, Faculté de Médecine R.T.H. Laennec, Lyon, France

**Keywords:** Human papillomavirus, Epstein-Barr virus, Cervical cancer, Uterine cervix, Confection

## Abstract

**Background:**

Despite the fact that the implication of human papillomavirus (HPV) in the carcinogenesis and prognosis of cervical cancer is well established, the impact of a co-infection with high risk HPV (HR-HPV) and Epstein-Barr virus (EBV) is still not fully understood.

**Methods:**

Fifty eight randomly selected cases of squamous cell carcinomas (SCC) of the uterine cervix, 14 normal cervices specimens, 21 CIN-2/3 and 16 CIN-1 cases were examined for EBV and HPV infections. Detection of HR-HPV specific sequences was carried out by PCR amplification using consensus primers of Manos and by Digene Hybrid Capture. The presence of EBV was revealed by amplifying a 660 bp specific EBV sequence of BALF1. mRNA expression of LMP-1 in one hand and protein levels of BARF-1, LMP-1 and EBNA-1 in the other hand were assessed by RT-PCR and immunoblotting and/or immunohischemistry respectively.

**Results:**

HR-HPV infection was found in patients with SCC (88%), low-grade (75%) and high grade (95%) lesions compared to only 14% of normal cervix cases. However, 69%, 12.5%, 38.1%, and 14% of SCC, CIN-1, CIN-2/3 and normal cervix tissues, respectively, were EBV infected. The highest co-infection (HR-HPV and EBV) was found in squamous cell carcinoma cases (67%). The latter cases showed 27% and 29% expression of EBV BARF-1 and LMP-1 oncogenes respectively.

**Conclusion:**

The high rate of HR-HPV and EBV co-infection in SCC suggests that EBV infection is incriminated in cervical cancer progression. This could be taken into account as bad prognosis in this type of cancer. However, the mode of action in dual infection in cervical oncogenesis needs further investigation.

## Background

Cervical cancer is the second most prevalent cancer among the Algerian women. The association between human papilomavirus (HPV) and cervical neoplasia is well documented [1]. High risk oncogenic HPV types (including HPV 16 and HPV 18) are associated with 99.7% of all low-grade cervical (CIN-1 or mild CIN) and high grade intraepithelial lesions (CIN-2/3) and hence, they play an important role in cervical cancer development. Now, and since 1976, it is well recognized that HPV infections in the cervix are frequently associated with intraepithelial neoplasia and invasive squamous cell carcinomas (SCC) with all their different histological variants (large-cell keratinizing, large-cell non-keratinizing and small-cell carcinoma).

The long period of time (years) it takes for the development of cervical cancer after HPV infection suggests the involvement of other etiologies (such as viruses or cell compounds) in malignancy process. The synergistic effect of carcinogenic factors such as two or more viruses interacting at different stages of tumor development has been reported [2-4]. Epstein-Barr virus (EBV), ubiquitous human gamma-herpes virus responsible for mononucleosis [5], could be one of the ‘helper’ viruses. It can be sexually transmitted [5] and replicates in cervix cells [6]. EBV infection, widely spread among the population [7,8], has been associated with an increasing number of lymphocytic and epithelial cancers, mainly Burkitt’s lymphoma, Hodgkin’s lymphoma, T cell lymphoma, nasopharyngeal carcinoma (NPC) and gastric adenocarcinoma [9,10].

BARF1 is one of the EBV-encoded proteins secreted in the serum of NPC patients [11] and expressed in more than 90% of NPC biopsies [12-15] and tumor epithelial cells of EBV-associated gastric carcinoma [12]. It has a malignant transforming activity in rodent fibroblasts [16] and in EBV-negative human B cells [14]. LMP1, another EBV oncogene candidate essential for B cell immortalization [17], was present in 30 to 50% of NPC biopsies [18]. This oncogene can activate a number of cellular key genes such as NFκB and EGFR [17,19]. LMP-1 can inhibit cell differentiation when transfected into epithelial cells [18].

Tseng et al. [20] reported a high incidence of EBV in lymphoepithelial like-carcinoma (LELC) patients but did not show any association with HPV. These findings are in contradiction with what has been previously reported [21,22]. Therefore, the oncogenic relationship between the two viruses remains not fully understood. Added to this, the presence of EBV in the cervix carcinoma remains equally a topic of great debate among virologists, confirmed by certain authors [2,23,24] but not by others [25,26].

As it is well known EBV can transform cells bearing the EBV/C3d receptor making them receptive to other oncogenic stimuli [27]. These receptors are widely detected on ecto- and endo-cervical biopsies of the uterine cervix [28-30]. EBV replicates in cervical epithelium and its possible role in cervical carcinoma development has been raised.

We looked, in this study, for the presence of both EBV and HPV DNA sequences in Algerian patients with SCC and cervical lesions. We examined the presence of EBV infection and the EBV-HPV co-infection. The presence of EBV in cervical cancer tissues suggests its possible involvement in the cervical cancer progression. Initially, PCR amplification was used to identify the co-infection. This was followed by an investigation on EBV oncogenes (BARF-1, LMP-1 and EBNA-1) expression using immunoblotting and immunohistochemistry. This expression would reflect the transformation mechanism of cervical cells.

Since EBV could play an important role in Nasopharyngeal carcinoma and Burkitt’s lymphoma highly frequent in Algeria, Our hypothesis was a possible co-infection by HPV and EBV in Algerian SCC and cervical lesions.

## Results

### Detection of HR-HPV and EBV in SCC biopsies

**Figure 1:**
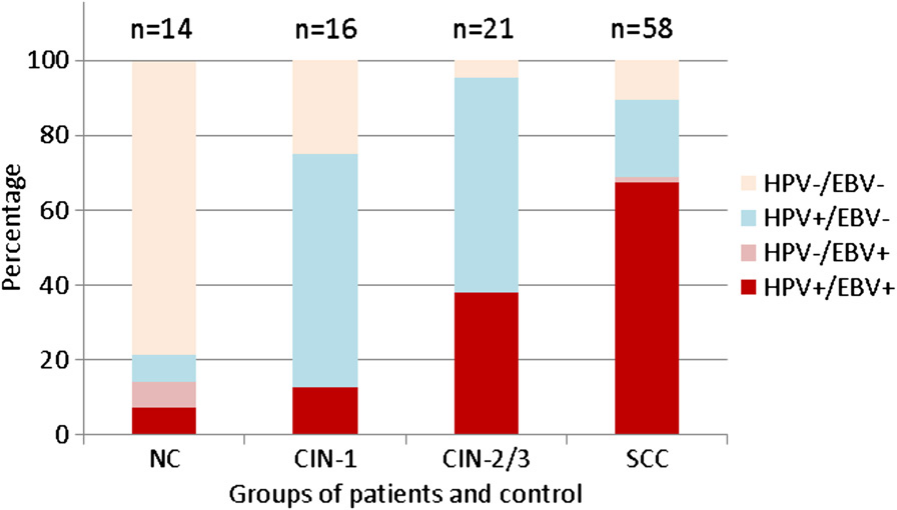
**Distribution of EBV and HR-HPV in patients with either cervical precancerous or cancerous lesions compared to normal subjects.** High risk human papillomavirus were detected by PCR (using consensus primers) and by Hybrid Capture 2 (Digene). Epstein-Barr virus was detected by PCR using primers that amplify the entire BALF-1 gene. Percentage in ordinate; Normal cervix, cervical lesions and carcinoma cases in Abscissa. NC: normal cervixes; CIN-2/3: High-grade cervical lesions; CIN-1: Low-grade cervical lesions; SCC: squamous cell carcinoma.

Fifty eight biopsies of SCC from Algerian women were analyzed to assess the presence of low and high-risk HPV strains. PCR and Hybrid Capture 2 (HC2) tests revealed that out the 58 studied samples 51 (88%) were positive to HR-HPV strains and 7 (12%) were HPV infection free. Among these HPV cases, 40 biopsies were exclusively infected by high-risk HPV strains and 11 were co-infected with high- and low-risk HPV (Figure 1).

**Figure 2:**
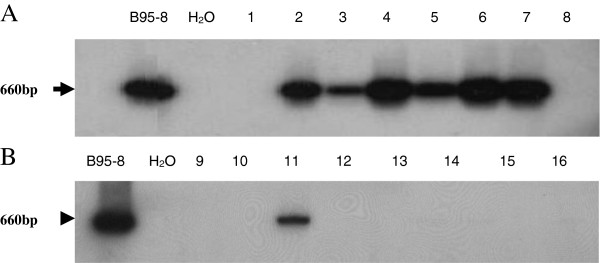
**Detection of EBV BALF1 gene by PCR.** Amplified PCR products were electrophoresed and the presence of BALF1 sequence [39] was confirmed by hybridization with ^32^P labeled BamH1-A fragment used as a probe. **A**: lanes 1-8 for tumor samples and **B**: lanes 9-16 for precancerous lesions. B95-8 DNA and H_2_O were used as positive and negative controls respectively.

In the same biopsies, the EBV genome was detected by PCR amplification of the entire EBV-encoded BALF1 gene. The PCR product (BALF1 sequence of 660 bp) was confirmed by Southern blot hybridization using the random primer kit with ^32^P-labeled BamH1-A fragment as a probe (Figure 2). As illustrated in Figure 1, 69% (40/58) cases of SCC cervical biopsies were positive to BALF1 sequence. EBV BALF-1 was present in only one case that was HPV negative. 67.2% (39/58) were co-infected with both viruses. 20.7% (12/58) were only HPV infected. Co-infections with HPV and EBV were detected more frequently in CIN-2/3 lesions and SCC groups than in NC and CIN-1 groups (p < 0.05). In terms of co-infection, there was no significant difference between NC and CIN-1 groups.

### PCR detection of HR-HPV and EBV in cervical lesions and in normal patients

Using PCR and HC2, 21 CIN-2/3 and 16 CIN-1 lesions and 14 normal tissue samples were investigated for HPV genome. The two methods showed a good correlation. 95% (20/21) CIN-2/3 cases were infected by HR-HPV compared to 40% (8/21) were co-infected with EBV. Among the 16 CIN-1 cases, HR-HPV was present in 75% (12/16) with 12.5% (2/16) of co-infections. The remaining twenty three EBV free cases (CIN-1 and CIN-2/3) were infected with either high or/and low risk HPV strains. Among the fourteen normal cervix samples, only two were HR-HPV positive and only one was co-infected (Figure 1).

### Transcriptional and translational expression of EBV in SCC cases

Limited by tissue specimens, the mRNA (LMP-1) and protein (BARF-1) expression of EBV oncogenes was assessed in only 23 out of 58 samples. However, all the three proteins (BARF-1, LMP-1 and EBNA-1) were immunohistochemically assessed in 45 out 58 of SCC biopsies (Table 1).

**Table 1:** Relationship between the detection of EBV oncoproteins (LMP-1 and BARF-1) and the presence of either or both HPV and EBV in malignant cervical biopsies

**EBV and HPV genomes detection by PCR: 23 SCC**	**LMP-1 detection by RT-PCR**	**BARF-1 detected by Western blotting**
14/23 HPV + EBV+	7 LMP1+	3 BARF1+
	7 LMP1-	2 BARF1+
8/23 HPV + EBV-	Negative	Negative
1/23 HPV-EBV+	Negative	Negative

**Figure 3:**
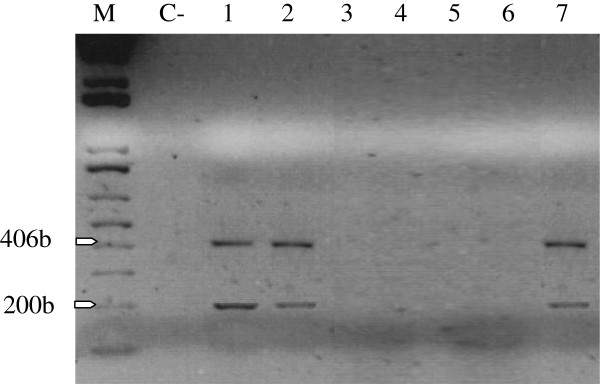
**Transcriptional expression of LMP-1.** A 406-bp PCR amplified fragment corresponding to the LMP-1 DNA sequence, and a 200-bp RT-PCR amplified fragment corresponding to the LMP-1 mRNA sequence. Lanes 1, 2 and 7 are positive signals. M: DNA fragments molecular markers. C-: negative control.

**Figure 4:**
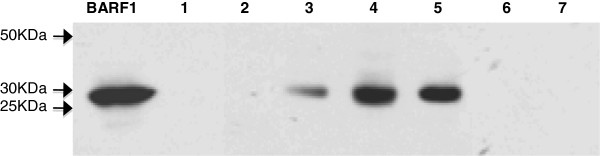
**Detection of BARF-1 protein by Western blotting.** Immunoblotting of BARF-1 on seven cervical tumor biopsies. Fifty micrograms of protein extract as measured by a Biorad protein assay were deposited onto 12% polyacrylamide gel and electrophoresed. Proteins were transferred onto nitrocellulose paper. BARF1 protein encoding 29 kDa (p29) was revealed by PepIII rabbit polyclonal anti-BARF-1. BARF-1 protein (p29) produced by the BARF-1 recombinant adenovirus system was used as a positive control indicated in first lane as BARF1.

**Figure 5:**
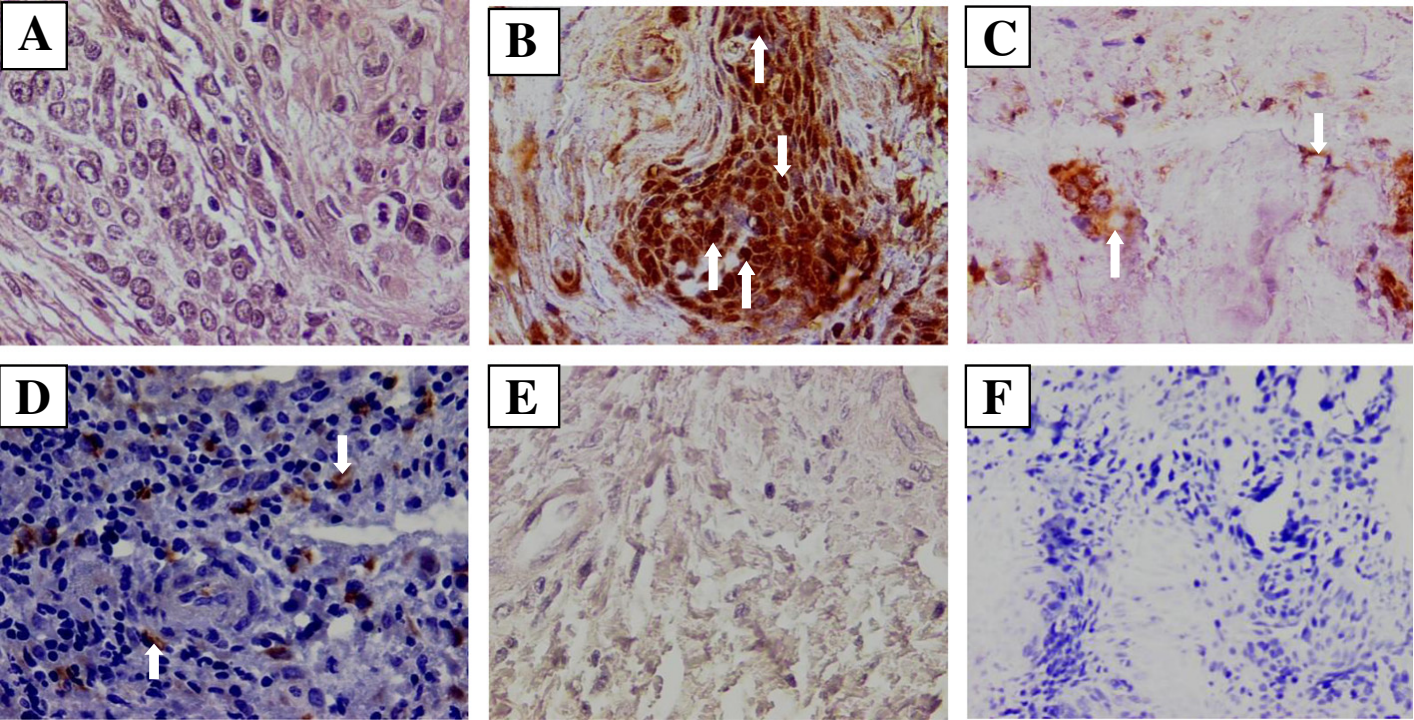
**Detection of HPV and EBV antigen by Immunohistochemistry.** Immunohistochemistry analysis was performed on sections of cervix tissue samples embedded in paraffin. HPV and EBV antigens were detected by peroxidase mediated DAB-staining (Brown). All samples were counterstained with hematoxylin-eosin and then observed under a light microscope (original magnification 200x). Positive response is marked with a white arrow. **A**: Squamous-cell carcinoma observed by heamatoxylin-eosine staining. **B**: Squamous carcinoma cells positive to EBNA-1 revealed by monoclonal anti-EBNA-1 antibody (OT-1). **C**: Squamous carcinoma cells positive to LMP-1 revealed by monoclonal anti- LMP-1 antibody (S12). **D**: Cytoplasmic and nuclear HPV staining with monoclonal antibodies (K1H8), which recognize HPV type 6, 11, 16, 18, 31, 33, 42, 51, 52, 56 and 58. **E**: Squamous-cell carcinoma negative to EBNA-1. **F**: Squamous-cell carcinoma negative to LMP-1. Weak staining of rare lymphocytes, the tumor cells are negative for LMP1.

As shown in Figure 3, the LMP-1 transcript was about 200 bp which was spliced from the 406 bp DNA sequence (see Material and Methods). Using immunoblotting technique, BARF-1 protein was detected in only 21.7% (5/23) of SCC biopsies analyzed from 50 μg cellular extract (Figure 4). We also examined the transcriptional and translational expression of HPV and EBV oncogenes (LMP-1 and BARF-1) by immunohistochemistry (IHC) (Figure 5). IHC analysis has allowed the identification of HPV and EBV products. As shown in Figure 5D, HPV was expressed in SCC biopsies. EBV antigens, EBNA-1 and LMP-1, were present as a brown nuclear and membrane staining (Figure 5B and C respectively). EBNA-1 protein was detected in 34.7% (8/23). While IHC analysis showed 26% (6/23) LMP-1 positive cases (Figure 5C). Negative squamous carcinoma cells for EBNA-1 and LMP-1 proteins are shown in Figures 5E and F respectively.

## Discussion

Our findings point out that most patients with either SCC or cervical lesions are HR-HPV infected. To our surprise, EBV-HPV co-infection was detected in the majority (67%) of cervical carcinoma among the Algerian women involved in this study. The presence of EBV was found in 69% of cervical cancer biopsies, suggesting its possible association with the development of cervical cancer. This is in agreement with previous reports [2,23,24]. However, other researchers, using in situ hybridization, reported controversial results [31-33]. Furthermore, Sasagawa et al. [24] demonstrated, by RNA in situ hybridization, the presence of EBV and the expression of EBV genes, EBER-1, LMP-1 and EBNA-1, in cervical biopsies.

Using immunohistochemistry technique, to our knowledge, we are the first to detect EBV antigens in the cervix tissue. The difference in our results between the PCR method and protein detection has three possible explanations; (i) DNA amplification using PCR is a more sensitive technique than the detection of antigens using Immunohistochemistry or immunoblotting, (ii) EBV genome is present but its genes are not abundantly expressed in EBV-infected cells and (iii) a contamination by EBV-positive B lymphocytes infiltrating the connective tissue or EBV-positive normal cervical epithelium adjacent to the lesions. PCR results on their own merely indicate the presence of EBV in the cervix tissue but not necessarily in the epithelial cells.

Our data suggest that co-infection with EBV and HR-HPV may be of cervical significance in the ethiopathogenesis of uterine cervical cancer. However, few cells in cervical tissue were infected by EBV and harbor EBV LMP-1 protein (Figure 5). The expression of LMP-1 and EBNA-1 in SCC may lead to the destruction of cancer cells as they are targets for EBV-specific cytotoxic T lymphocytes. Such process has been reported in gastric [12] and Burkitt lymphomas [10]. However, the cervix is histologically different from lymphoid tissues which are localized very far from the cervical epithelium by thick myometrial tissue of the cervix. The same situation has been observed in HPV infection where E6-E7 oncogenes are frequently expressed in cervical neoplastic lesions and which are equally targets for cytotoxic T lymphocyte response in patients with cervical neoplasia [34].

EBV DNA has been shown in the exfoliating cells [28]. The virus could infect cultured ectocervical cells and express its late antigens suggesting an association between EBV replication and epithelial differentiation [35]. Infectious EBV able to transform B lymphocytes has been isolated from cervical washes from women recovering from EBV mononucleosis infection or EBV-seropositive with no acute infection. Several studies showed that EBV infection is sexually transmitted targeting the uterine cervix [5,36-38].

In this study, EBV latent genes were expressed in some squamous epithelia. The cervix might be a site for chronic EBV shedding in a similar manner to the nasopharynx. It is widely accepted that EBV infects B lymphocytes through CD-21 (EBV/C3d) receptor [27] which are equally present in cervix cells. The squamocolumnar junction of the cervix is the area where tissue repair and inflammation frequently occur, and where cervical malignancies associated with HPV infection develop. Similarly, chronic cervicitis also may help the EBV infection [4]. The low rate of EBV infection in normal cervixes and in pre-cancer lesions compared with SCC cases of patients used in this study suggests that EBV may play a role in late cervical carcinogenesis. Further investigation is required to clarify whether the presence of EBV is a worse prognosis for the development of cervical cancer. EBV may be acting as a cofactor with another carcinogenic agent(s), possibly human papillomaviruses, in the final invasive cancer progression.

## Conclusion

Preliminary presented results of EBV and HR-HPV co-infection in Irish, North American, Thailand and Japanese SCC cases were recently reported [2,3]. In our hands we showed that most Algerian SCC patients were HPV-EBV co-infected. A lesser degree of co-infection was observed in pre-cancerous lesions of the cervix. A possible joint effect of the two viruses on cervix tumor development should be considered. Such hypothesis is strengthened by EBV oncogene expression. It is clear that the presence of EBV and its relationship with HPV in cervical oncogenesis need to be further investigated.

## Materials and methods

### Samples

**Figure 6:**
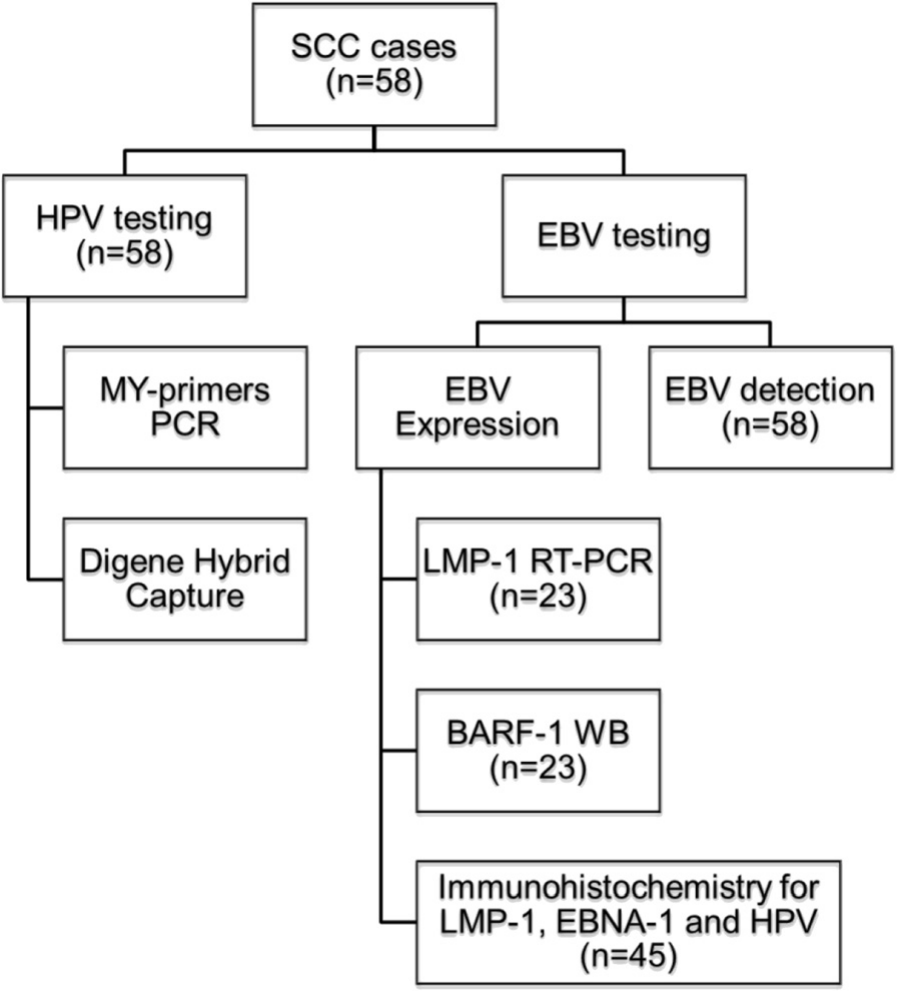
**Flow chart showing the organization of HPV and EBV detection and expression in squamous cell carcinoma (SCC) cases by PCR using MY consensus primers and by Digene Hybrid Capture II (HC II).** The Epstein-Barr virus (EBV) was detected by PCR using primers that amplify the entire BALF-1 gene. EBV expression was investigated by LMP-1 Reverse Transcription-PCR, BARF-1 Immunoblotting and LMP-1 and EBNA-1 Immunohistochemistry.

One hundred and nine cervix specimens taken from female patients attending a gynecological department in Algiers (Algeria) were randomly selected and enrolled for EBV and HPV-DNA detection and genotyping (Figure 6). We obtained from the Scientific Council of the Faculty of Natural Sciences and Life, University Setif-1 (Algeria) permission to investigate on human samples, as required by the Helsinki declaration respecting ethical principles for medical research, including research on identifiable human samples and data (Ref: CSF/SNV/2013). Patients were asked to participate in the study, and informed consent was obtained for HPV testing. They were classified as follows: Group 1 was composed of 58 patients having invasive or in situ squamous-cell carcinoma (SCC) of the uterine cervix. When the tumor became evident, biopsies were freshly obtained from these women immediately after surgery and were snap-frozen in liquid nitrogen, transported to the laboratory and then stored at −80°C until processed. Histological grading was performed by an experienced pathologist. Each biopsy was cut with sterile blades, homogenized and divided into three fractions; used respectively for RNA, DNA, and protein preparations. Group 2 were 16 patients with low-grade lesions (Cervical intraepithelial neoplasia-1 (CIN-1), condyloma, and cervicitis). Group 3 were 21 women with CIN-2/3 (known as mild and severe dysplasia). The samples of groups 2 and 3 were collected from the apparently malignant zone. The samples were then placed into a one ml tube of the transport medium (ViraPap/Viratype transport medium, (Digene, Silver Spring, MD)) and stored at −20°C until processing. Group 4 were 14 cases of normal cervixes obtained by scraping the junction area of healthy women undergoing routine cervical screening. Conventional cytology was performed for this group.

### DNA extraction and purification

Tissues were cut using sterile blades in a lysis buffer (10 mm Tris, pH 7.4, 50 mm EDTA, 150 mm NaCl, 1% sarcosyl) and treated with RNase A (10 mg/ml) for two hours at 37°C. DNA was obtained after proteinase K digestion, by phenol-chloroform extraction, ethanol precipitation and solubilized in 20 μl TE buffer (10 mm Tris–HCl, pH 8.0; 1 mm EDTA). DNA quantities and purity were assessed by spectroscopy.

### Hybrid Capture 2 (HC2)

The HC2, a standardized US FDA-approved test has been employed to detect one or more of 13 carcinogenic HPV types (16, 18, 31, 33, 35, 39, 45, 51, 52, 56, 58, 59 and 68). This enzyme-linked immunosorbent assay is based on a sandwich hybridization followed by a non-radioactive alkaline phosphatase reaction following the manufacturer’s instructions.

### Polymerase chain reaction for detection of HPV DNA and β-globin

Specimens were subjected to PCR with general HPV primers using conventional L1 consensus primers, MY11 (GCMCAGGGWCATAAYAATGG) and MY09 (CGTCCMARRGGAWACTGATC). The assays were conducted according to the manufacturer’s protocol (Digene SHARP Signal™ System).

### EBV-DNA identification by Southern blotting

As previously published by our group [39], EBV PCR was performed in 25 μl reaction mixture containing Taq polymerase buffer (10 mM Tris–HCl, pH 8.3; 50 mM KCl), 5 mM MgCl2, 200 mM of each deoxyribonucleic triphosphate, and 250 nM of each primer and 1 microgram of DNA sample. The whole BALF1 sequence was amplified from the DNA extracts using two primers: ALF1-S (GGGGATCCAATGAACCTGGCCATTGCTCTG) (upstream) at position 165,517 of EBV genome and ALF1-AS (CGGAATTCTTACAAAGATTTCAGGAAGTC) (downstream) at position 164,858. These primers permitted to amplify a fragment of 660 bp (the entire BALF-1 gene). Ten microliters of the amplified fragment were loaded onto 2% agarose gel, electrophoretically separated and revealed by ethidium bromide and then transferred onto reinforced nitrocellulose membranes (Schleicher & Schuell, Germany). Amplified fragment was detected by hybridization using ^32^P-labeled-BamH1-A probe prepared with a random primer DNA-labeling kit (Stratagene) [33]. The hybridization was carried out in a modified solution described by Ausubel et al. [34] with 10^**6**^ cpm/ml of the labeled BALF1 probe.

### RT-PCR

RNA was extracted from control and specimens with Trizol reagent (Invitrogen) according to the manufacturer’s instruction. Total RNA was treated with DNase (amplification grade Deoxyribonuclease I, Invitrogen). Reverse transcriptase PCR (RT-PCR) was carried out as previously described [10]. LMP-1 was amplified with primers 5′-CGGGATCCATGGAACGCGACCTTGAGAG and 5′-CGGGATCCCAACAGAAGAGACCTTCTCT.

### Western blotting analysis (immunoblotting)

Biopsy tissues were cut using sterile blades in lysis buffer, RIPA (650 mM NaCl, 5 mM EDTA, 20 mM Tris pH 7.5, 1% Triton, 0.5% and 0.1% SDS), and treated with antiprotease [11]. The lysates were sonicated and stored at −80°C. Lysates were first adjusted to containing equal amounts of proteins (50 micrograms) measured by a Biorad protein assay (Bio-Rad Laboratories, Inc.) then diluted with one volume of gel sample buffer (0.2% Bromophenol blue, 4% SDS, 200 mM DTT (dithiothreitol), 20% glycerol, 125 mM Tris–HCl, pH 6.8) and boiled for 5 min. Protein samples were separated on 12% polyacrylamide denaturing gels and electrophoretically blotted onto reinforced nitrocellulose as previously described. After transfer, the filters were subsequently incubated overnight at 4°C with anti-BARF-1 (anti-Pep 2A) [10]. The filters were then washed and incubated for 1–2 hours at room temperature with peroxidase labeled polyclonal anti-rabbit anti-human immunoglobulin as a secondary antibody. The antigen-antibody complexes were visualized using an enhanced chemiluminescence system (ECL; Amersham) as instructed by the manufacturer.

### Immunohistochemical staining

Cervix tissues were formalin fixed, dehydrated in alcohol and embedded in paraffin. Immunostaining was performed according to the streptavidin-biotin peroxidase complex method, using monoclonal antibodies against HPV, LMP-1 and EBNA-1. Briefly, sections were cut to a thickness of 4 μm, mounted on silane-treated slides (Superfrost), and dried 1 hour at 60°C. All sections were then deparaffinized in xylene, rehydrated through alcohol, and washed in phosphate-buffered saline. This buffer was used for all subsequent washes. Sections for LMP-1, EBNA-1 and HPV detection were heated in a microwave oven twice for 5 min in citrate buffer (pH 6.0). Mouse monoclonal antibodies (at a dilution of 1:500) anti-LMP-1 (S12, BD Biosciences Pharmingen), and anti-EBNA-1 (sc-29; Santa Cruz; Germany) were used respectively as the primary antibody and incubated overnight at room temperature followed by a conventional streptavidin peroxidase method (DAKO, Denmark). Signals were developed with 3, 30-diaminobenzidine for 5 minutes and counter-stained with hematoxylin. The results were scored for the positive signals in tissue cells. The same samples were sectioned at 4 μm thickness and routine hematoxylin- and eosine-stained for histologic examination.

## Abbreviations

HPV: Human papillomavirus; EBV: Epstein-Barr virus; SCC: Squamous-cell carcinoma; CIN: Cervical intraepithelial neoplasia.

## Competing interests

The authors declare that they have no competing interests.

## Authors’ contributions

AK for HPV classification and HPV detection, RT-PCR, PCR, Immunohistochemistry, Southern blotting, immunoblotting, analysis of results and redaction. NS for HPV classification and HPV detection. AB for preparation of cell scrapings and biopsies of the cervix. KH for analysis of results. AG for preparation of cell scrapings and biopsies of the cervix. TO for analysis of results and for redaction. AB for analysis of results and for redaction. All authors read and approved the final manuscript.
